# The Recombinant Form of *Trypanosoma cruzi* P21 Controls Infection by Modulating Host Immune Response

**DOI:** 10.3389/fimmu.2020.01010

**Published:** 2020-06-05

**Authors:** Flávia Alves Martins, Marlus Alves dos Santos, Júlia de Gouveia Santos, Aline Alves da Silva, Bruna Cristina Borges, Mylla Spirandelli da Costa, Paula Cristina Brígido Tavares, Samuel Cota Teixeira, Rebecca Tavares e Silva Brígido, Thaise Lara Teixeira, Cassiano Costa Rodrigues, Nadjania Saraiva de Lira Silva, Rayane Cristina de Oliveira, Laura Caroline de Faria, Marcela Rezende Lemes, Renata Graciele Zanon, Tatiana Carla Tomiosso, Juliana Reis Machado, Marcos Vinicius da Silva, Carlo José Freire Oliveira, Claudio Vieira da Silva

**Affiliations:** ^1^Laboratório de Tripanosomatídeos, Departamento de Imunologia, Instituto de Ciências Biomédicas, Universidade Federal de Uberlândia, Uberlândia, Brazil; ^2^Departamento de Microbiologia, Imunologia e Parasitologia, Universidade Federal de São Paulo, São Paulo, Brazil; ^3^Departamento de Microbiologia, Imunologia e Parasitologia, Universidade Federal do Triângulo Mineiro, Uberaba, Brazil; ^4^Departamento de Anatomia Humana, Instituto de Ciências Biomédicas, Universidade Federal de Uberlândia, Uberlândia, Brazil; ^5^Setor de Histologia, Instituto de Ciências Biomédicas, Universidade Federal de Uberlândia, Uberlândia, Brazil; ^6^Departamento de Patologia, Genética e Evolução, Universidade Federal do Triangulo Mineiro, Uberaba, Brazil

**Keywords:** Chagas disease, P21, acute experimental infection, immune evasion, intracellular replication

## Abstract

*Trypanosoma cruzi* P21 protein (P21) is a putative secreted and immunomodulatory molecule with potent bioactive properties such as induction of phagocytosis and actin cytoskeleton polymerization. Despite the bioactive properties described so far, the action of P21 on parasite replication in muscle cell lineage or *T. cruzi* parasitism during acute experimental infection is unclear. We observed that recombinant P21 (rP21) decreased the multiplication of *T. cruzi* in C2C12 myoblasts, phenomenon associated with greater actin polymerization and IFN-γ and IL-4 higher expression. During experimental infection, lower cardiac nests, inflammatory infiltrate and fibrosis were observed in mice infected and treated with rP21. These results were correlated with large expression of IFN-γ counterbalanced by high levels of IL-10, which was consistent with the lower cardiac tissue injury found in these mice. We have also observed that upon stress, such as that induced by the presence of the IFN-γ cytokine, *T. cruzi* produced more P21. The effect of P21 in controlling the replication of *T. cruzi*, may indicate an evolutionary mechanism of survival developed by the parasite. Thus, when subjected to different stress conditions, the protozoan produces more P21, which induces *T. cruzi* latency in the host organism, enabling the protozoan to evade the host's immune system.

## Introduction

Chagas disease is a neglected tropical disease caused by the flagellated protozoan *Trypanosoma cruzi* (*T. cruzi*). It was discovered by Carlos Chagas, a physician who described in detail the parasite, its vectors and hosts (1, 2). In Latin America, it is estimated that about 20 million people are at risk of infection. Worldwide, Chagas disease affects 8 million people and causes approximately 10,000 deaths every year, impacting the economy, public health and quality of life of patients affected by the disease (3, 4).

During the acute phase of Chagas disease cardiac changes occur in the first weeks after parasite inoculation and usually last from weeks to a few months (5). After a few weeks, the adaptive immune response develops in the host, the number of parasites decreases dramatically, pro-resolving mediators usually induce resolution of myocardial inflammation. Residual abnormalities of acute myocarditis include tissue fibrosis and cardiomyocyte hypertrophy (6). Several studies have focused on the *T. cruzi* evasion and the mechanisms by which this parasite keeps in dormancy within its hosts, especially in muscular and cardiac cells.

In these studies, it has been shown that membrane or secreted *T. cruzi* molecules are responsible for these effects. For example, the interaction between the parasite proteins and their host cell molecular targets promotes *T. cruzi* cell invasion and survival (7). Tc-85, Tc-1, gp83 glicoprotein, cruzipain and oligopetidase B are trypomastigote proteins involved with host cell invasion, while the ones expressed by metacyclic tripomastigotes are gp82 and gp35/50 (8–10). Gp85/trans-sialidase glycoproteins interact with laminin and cytokeratin fostering parasite host cell adhesion and invasion (11–13). *T. cruzi* mucins (TcMUC) play a role on parasite recognition and invasion, protecting the parasite from host defense mechanisms (7, 14). Cruzipain (Cz) is a surface *T. cruzi* protease involved on the survival, growth, cellular differentiation and internalization of the parasite (15, 16). This protein has an important role on parasite escape from phagolysosomes. It also stimulates arginase activity, which results in anti-inflammatory process with enhanced phagocytic capacity and diminished killing functions (7, 15, 17).

Besides these previous molecules, Silva et al. characterized a 21 kDa ubiquitous protein secreted by *T. cruzi*, the P21. Since then, our group of research conducted several studies using the recombinant form of P21 (rP21) in order to evaluate the potential biological activities of native molecule. We observed that rP21 also promoted cellular invasion by the parasite (10), presented chemotactic activity and induced actin cytoskeleton polymerization by binding to the CXCR4 receptor (18).

*In vivo* administration of rP21 into unstimulated control mice induced myeloperoxidase and IL-4 production and decreased the formation of blood vessels (19). The anti-angiogenic role of rP21 was shown by the recruitment of leukocytes into the inflamed tissue and induction of IL-4 production. This led to a polarization of macrophages into the M2 profile through increased sFlt-1 expression and decreased production of Flt-1, ezrin, FAP1, AFAPL1, and moesin (20). The effects of P21 on muscle or cardiac cells and on the acute experimental infection has not even been investigated. In this study, we evaluated the impact of rP21 on the course of *in vivo* acute experimental infection with *T. cruzi* as well as its effect on the parasite multiplication *in vitro* on muscle cell lineage.

## Materials and Methods

### Cells and Parasites

C2C12 (murine myoblast) and Vero cells were cultured in Dulbecco's Modified Eagle Medium (DMEM) supplemented with 10% fetal bovine serum (FBS, Cultilab), 10 mg/mL streptomycin (Sigma), 100 U/mL penicillin (Sigma), and 40 mg/mL gentamycin (Sigma) at 37°C in a humidified atmosphere containing 5% CO_2_. To generate tissue-cultured trypomastigotes (TCT) from Y and G strains, confluent monolayers of Vero cells were infected with metacyclic trypomastigotes and maintained for at least 2 weeks to establish the intracellular cycle.

### Animals and Ethics

Six-to-eight weeks old male BALB/c mice were maintained under standard conditions on a 12 h light-dark cycle in a temperature-controlled setting (25°C), with food and water *ad-libitum*. Maintenance and care of animals complied with the guidelines of the Ethics Committee for the Use of Animals (CEUA). Animal euthanasia was performed based on international welfare grounds according to the American Veterinary Medical Association Guidelines on Euthanasia. This study was approved by CEUA-UFU, with protocol number 087/15.

### rP21 Purification

For production of recombinant P21 protein, *Escherichia coli* BL21 strain transfected with plasmid pET-28th (+) (Novage) using the gene coding for rP21 (GenBank: EU004210.1) were used. In a pre-inoculum, the bacteria were placed in Luria-Bertani (LB) medium with the Kanamycin selection antibiotic (50 μg/mL). The pre-inoculum was kept under stirring for 18 h at 37°C. Subsequently, the pre-inoculum was diluted 1:50 in the same medium and incubated at 37°C, with shaking at 150 RPM, until reaching the optical density (OD) of 0.6 to 0.9. Then 1 mM Isopropyl β-D-1-thiogalactopyranoside (IPTG) was added to stimulate expression of the recombinant protein. After 3 h incubation, the medium containing the bacteria was centrifuged at 10,000 × g for 20 min, the supernatant discarded, and the precipitate resuspended in PBS. To promote bacterial lysis, 10 μL of lysozyme (50 mg/mL) was added to the resuspended material every 10 mL of solution for 20 min. Then the Sonicador Branson Sonifier 450 was used for 20 1-min cycles with 30-s interval between cycles. The bacterial lysate was centrifuged at 20,200 × g for 20 min at 4°C. The supernatant was discarded, and the pellet resuspended in 6 M urea buffer solution. The sample was incubated with a nickel resin and allowed to stir overnight at 4°C. The following day, the resin was subjected to: (1) three washes in binding buffer (5 mM imidazole, 500 mM NaCl, Tris-HCl 20 mM pH 8.0, 6 M urea); (2) three washes in wash buffer (20 mM imidazole, 500 mM NaCl, 20 mM Tris-HCl pH 8.0, 6 M urea); (3) four elutions with elution buffer (1 M imidazole, 50 mM NaCl, 20 mM Tris-HCl pH 8.0, 6 M urea). Dialysis was performed for 48 h under continuous stirring at 4°C with a micropore dialysis membrane (3.5 kDa/Spectra), the sample concentration was quantified by Bradford technique (21). rP21 eluted purity was analyzed by 13% SDS-PAGE gel with Coomassie Blue staining.

### F-Actin Staining

C2C12 cells were seeded at a density of 2 × 10^4^ cells/coverslip in 24-well micro-plates. After adhesion, cells were treated or not with rP21 (40 μg/mL) (18). After 96 h, cells were fixed with 4% formaldehyde, washed 3 times and then left in PBS. F-actin staining was performed using TRITC-phalloidin diluted 1:1000 in PBS+saponin (0.01%). After washes, the coverslips were mounted on glass slides and images captured with a 63 × oil immersion objective using an inverted fluorescence microscope (Zeiss Axiovert 200 M). Digital images were analyzed using confocal fluorescence microscopy software (Zeiss, LSM 510 Meta, Germany). The mean F-actin fluorescence was determined by setting a high threshold in ImageJ software (National Institutes of Health, USA). For flow cytometry analysis, samples were acquired in Guava easyCyte Flow Cytometer and results analyzed with Guava® Suite Software 2.7.

### *In vitro* Multiplication Assay

C2C12 myoblasts were seeded onto 24 well-plate (10^5^ cells/well) containing 13 mm round coverslips and left overnight. Afterwards, cells were infected with tissue culture derived trypomastigote forms of the Y or G strains of *T. cruzi* (TCT Y/ TCT G; 10 parasites/cell) for 3 h. Cells were washed three times with PBS to remove non-internalized parasites followed by incubation with medium containing or not rP21 (40 μg/mL). Cell culture medium was changed after 48 h. At 96 h, cells were fixed on formaldehyde solution and incubated with anti-*T. cruzi* polyclonal antibody diluted 1:200 (v/v) in PBS solution containing 0.15% gelatin, 0.1% sodium azide, and 1% saponin (PGN-Saponin). Then, cells were incubated for 1 h with FITC-conjugated anti-rabbit IgG diluted 1:200 in PGN-Saponin containing also 500 ng/ml phalloidin-TRITC for F-actin staining and 10 μg/mL DAPI. The coverslips were mounted on glass slides and images captured with a 63 × oil immersion objective using an inverted fluorescence microscope (Zeiss Axiovert 200 M). The number of parasites per cell was quantified in 100 infected cells.

### FACS Analysis

C2C12 cells were plated into 6-well-plates (10^6^ cells/well) and incubated at 37°C/5% CO_2_ overnight. Cells were infected, fixed and stained as described in the previous item. The quantification of infection was performed by flow cytometry. All samples were acquired in a flow cytometer (FACSCantoII Becton, Dickinson, and Company – BD, Franklin Lakes, NJ, USA) with at least 50.000 events acquired per sample, in triplicates. Results were analyzed using the FlowJo X software (Tree Star Inc., Ashland, OR, USA).

### Measurement of Nitrite (NO^2−^)

For nitrite (NO^2−^) quantification, we used the colorimetric Griess reaction. Briefly, 50 μL of the culture supernatant from infected cells under different treatments was added to a 96-well-plate, followed by the same volume as the Griess reagent. This is composed of 1% sulfanilamide and N-(1-naphthyl) ethylenediamine hydrochloride (NEED), both diluted in 2.5% H_3_PO_4_ solution. To make a standard curve, a sodium nitrite solution at an initial concentration of 200 μM was diluted serially at a factor of 2 in the DMEM medium (also used as blank), until it reached a dilution of 0.39 μM. After a 10-min incubation in the dark, a reading on the spectrophotometer was performed at 540 nm. The absorption of the different samples was compared with a standard curve, and the results obtained were expressed as the mean of the triplicate ± standard deviation.

### Viability Analysis of Intracellular Amastigotes

C2C12 myoblasts were plated, infected with TCT Y and treated as previously described. After 96 h, cells were lised and intracellular amastigotes were recovered with insulin syringes coupled with needles of 4.0 mm long x 0.23 mm wide. These parasites were analyzed for their viability and ability to differentiate into epimastigotes by incubation in LIT medium pH 7.2 for 10 days. After this time, the parasites were counted in a Neubauer chamber and the number of epimastigotes x 10^4^ was graphically expressed.

### ELISA Assays to P21 Detection

Indirect ELISA was developed to detect P21 in infected cells or in epimastigotes extract. Microplates were coated with 50 μL of pre-dosed and normalized crude cell extract (50 μg/mL) in 0.06 M carbonate-bicarbonate buffer (pH 9.6) and incubated overnight at 4°C. After washes, 25 μL of serum from immunized rabbit (1:200) was added to the plates and incubated for 1 h at 37°C. Subsequently, the secondary antibody HRP-labeled goat anti-rabbit IgG (A0545 Sigma Aldrich) was added to the plates. The reaction was revealed by addition of enzyme substrate (0.03% H_2_O_2_ and o-phenylenediamine in citrate-phosphate buffer 0.1 M, pH 5.0) and stopped by addition of 2N H_2_SO_4_. The optical density (OD) was determined at 492 nm. rP21 was used for the preparation of the standard curve with initial concentration of 200 μg/mL.

### Experimental Infection

Mice were randomized into four groups with 10 animals per group: PBS non-infected; rP21 non-infected; PBS infected and rP21 infected. They were inoculated subcutaneously with 10^6^ or 10^5^ bloodstream trypomastigote forms of the Y strain of *T. cruzi*. rP21 (100 μg/animal) or PBS treatment were administered subcutaneously at days 0, 5 and 15 post-infection. At day 21 post-infection, animals were euthanized under anesthesia. Blood, heart and spleen samples were collected for morphological, immunological and molecular analyses.

### Histological Analysis

Heart samples were fixed in 10% buffered formalin solution, dehydrated in ethanol solution, diaphanized in xylene and embedded in paraffin. Blocks containing hearts were sectioned at 5 μm thick sections, and then placed onto glass slides and stained.

#### Hematoxylin and Eosin (HE) Staining

To evaluate the number of amastigote nests and blood vessels, inflammatory infiltrate and damage tissue score, slides of cardiac tissue were stained with hematoxylin and eosin (HE). The amastigote nests and number of blood vessels in each slide were quantified under light microscopy, normalized and plotted as blood vessels or amastigote nests/μm^2^ of heart tissue. Inflammatory infiltrate and damage tissue were scored by intensity: (−) absent, (+) mild, (++) moderate, (+ + +) intense as described by Silva et al. (22).

#### Picrossirius Staining

Five-micrometer thick heart tissue sections were subjected to successive immersions in xylene, hydrated in decreasing ethanol and water concentrations, and stained with picrossirius solution (Sigma Aldrich) for 50 min, followed by washes in distilled water. Then, sections were placed in hematoxylin solution for 4 min, washed in water, and stained for 1 min with aqueous eosin. After dehydration in ethanol and xylene diaphanization, slides were mounted with Entellan®. The quantification of total collagen fibers was performed using ImageJ software; images of 20 fields of each sample were taken with a light microscope (Nikon). To measure the % (percentage) of type I and type III collagen, the ImageJ software were calibrated through the Split Channel option and Threshold, where all images were adjusted to capture the same color range. The percentage of each type of collagen per image was tabulated for analysis in GraphPad Prism 6.01. A total of 20 fields were analyzed per sample.

#### Toluidine Blue Staining

To measure the number of recruited mast cells in cardiac tissue, heart tissue sections were stained with toluidine blue. Briefly, xylene deparaffinized sections were rehydrated by a rise in 96% ethanol followed by 10 min in 70% ethanol and 3 rinses in ultrapure water. Fully hydrated sections were then placed in phosphate-citrate buffer pH 3.0 for 5 min and stained with 0.5% toluidine blue for 3 min. Excess dye was removed by dipping the slides in buffer. Samples were clarified by increasing ethanol and xylene concentrations, and slides were mounted with Entellan®. Data were analyzed using the ratio of degranulated, granulated and total number of mast cells per area count (total number of mast cells/mm^2^).

### Quantitative Real Time PCR (qPCR)

The parasite load was estimated by the quantification of *T. cruzi* DNA in heart samples. Total genomic DNA was extracted from control mice and those infected with *T. cruzi* using PureLink® Genomic DNA Kit (Invitrogen, Carlsbad, CA, USA), according to manufacturer's protocol. The standard curve was performed with serial dilutions of DNA obtained from 1 × 10^8^ *T. cruzi*. The concentration and quality of DNA was determined at 260/280 nm. For DNA amplification, the following pair of primers P21fw (5-AACGCCACCATCAATCTTTTG−3) and P21rv (5-CGTCGCATTCCTCATTTCTTC-3), and probe P21p (5-ACGCCATCGTCATGTGCGCAG-3) were used, resulting in the amplification of a 65 bp fragment of *T. cruzi* genomic DNA (XM 812182.1). qPCR reactions were performed with a final volume of 12.5 μL containing 2 μL (50 ng) of the DNA extracted from heart samples. Reactions were processed in ABI7300 equipment (Applied Biosystems) under the following conditions: 50°C for 2 min, 90°C for 10 min, 40 cycles at 95°C for 15 s and 60°C for 45 s and 72°C for 30 s. After the final elongation of qPCR, samples were submitted to temperature variation from 50 to 95°C, with a gradual increase of 0.5°C/s to obtain the melting temperature (Tm) and non-specific products.

### Cytokine Immunoassays

For evaluation of cytokine levels in cardiac and spleen tissues harvested from control and *T. cruzi* infected mice, tissue sections were immersed in PBS solution containing protease inhibitor cocktail (1 tablet diluted in 50 ml of PBS-Complete, Sigma Aldrich). After homogenization with tissue homogenizer, the quantification of cytokines and total proteins was performed. In addition, serum from infected or uninfected mice and supernatant of infected cells (harvested at 96 h post-infection) were also used for evaluation of cytokine production. Levels of IFN-γ, TNF-α, IL-10 and IL-4 were measured with BD OptEIA ELISA kit.

### Indirect Immunofluorescence

Paraffin-embedded heart samples were used for indirect immunofluorescence as follows: after removal of paraffin with xylene and alcohol, samples were treated with 50 mM ammonium chloride for 1 h, and blocked with albumin (one egg white qsq in 100 ml of distilled water) for 20 min and with skimmed milk overnight. Then, they were incubated overnight with rabbit polyclonal antibody specific to rP21 and mouse polyclonal antibody specific to *T. cruzi* diluted in PGN-saponin (PBS + gelatin + azide) (1:100). The final step was an incubation for 1 h with mouse anti-rabbit IgG Alexa Fluor® 488 (Thermo Fisher Scientific, USA) conjugated antibody (1:200) and rat anti-mouse IgG TRITC-conjugated antibody (1:200) diluted in PGN-saponin. Samples were analyzed with a confocal microscope.

### RNA Extraction, cDNA Synthesis, and RT-qPCR

RiboZolTM Plus RNA Purification Kit (Amresco) was used for RNA extraction according to manufacturer's recommendations. The concentration and quality of RNA was determined at 260/280 nm. The High Capacity cDNA Reverse Transcription Kit (Applied Biosystems) was applied for the reverse transcription reaction according to the manufacturer's recommendations. Only samples with an OD260/280 ratio from 2.0 ± 0.1 were analyzed. The RT-PCR was performed with 2 g RNA in a final volume of 20 uL under the following conditions: 25°C for 10 min, followed by 37°C for 120 min. Afterwards, samples were heated up to 85°C for 5 min and finally cooled to 4°C. cDNA was stored at−20°C. The gene quantification was determined in ABI 7300 equipment (Applied Biosystems). Standard cycling conditions were used as recommended by the manufacturer: 95°C for 10 min, (95°C for 15 s, 60°C for 1 min) × 40 cycles, and the melt curve analysis at 95°C for 15 s, then 60°C for 1 min. Each PCR reaction was conducted in triplicate. In addition, melting curve analysis was performed in each assay for detection of non-specific amplification. The relative levels of gene expression were analyzed with 2-Ct method, where Ct = Ct infected group (Ct gene target - Ct gene endogenous) - Ct non-infected group (Ct gene target - Ct gene endogenous). The D71/D72 was used as reference gene (endogenous).

### Statistical Analysis

Data are expressed as mean ± standard deviation of experiments performed at least three times in triplicate. All data were first checked for normal distribution. Significant differences were determined by one-way ANOVA, Tukey's multiple comparisons test and Student's *t*-test (two-sided) for the parametric data or the Mann-Whitney test for non-parametric data according to the experimental design (GraphPad Prism Software version 6.01). Data were considered statistically significant at *p* < 0.05.

## Results and Discussion

### rP21 Enhances Actin Polymerization in C2C12 Cells and Controls *in vitro T. cruzi* Multiplication

**Figure 1:**
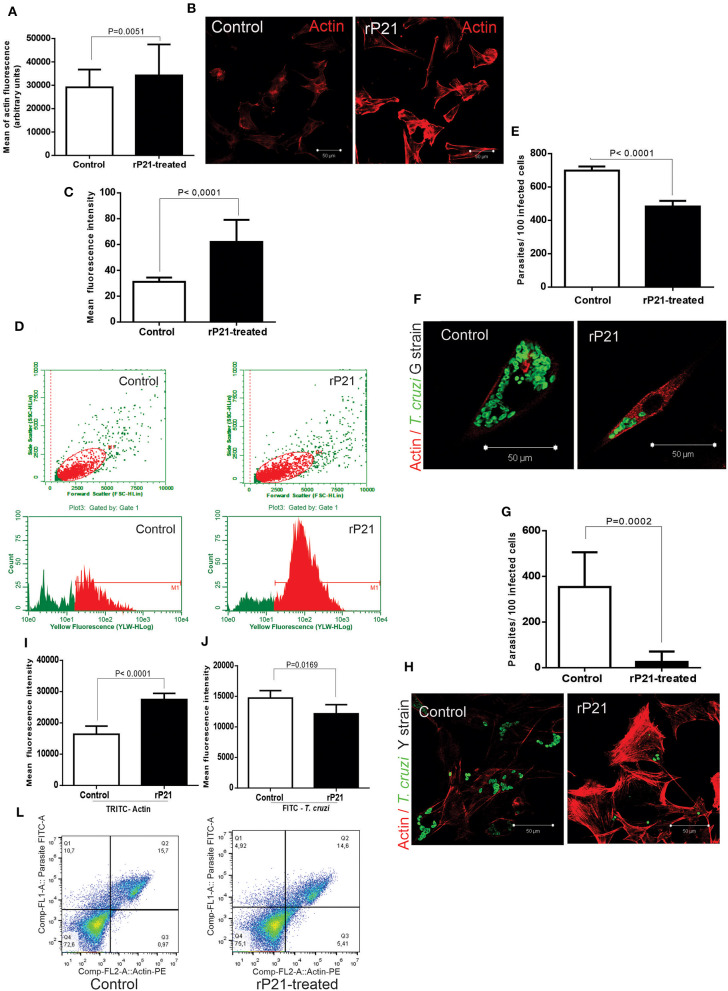
rP21 enhances actin polymerization of C2C12 myoblasts and impairs *T. cruzi* intracellular replication. **(A)** rP21 induces actin polymerization in C2C12 cells after 96 h treatment. F-actin is stained with TRITC-phalloidin (red). **(B)** Representative images of F-actin staining are shown. **(C,D)** Flow cytometry analysis (FACS) confirmed the enhancement of actin polymerization when C2C12 cells were treated with rP21. **(E–H)** rP21 leads to impaired multiplication of the G **(E)** and Y **(G)** strains. Parasites (green) are labeled with FITC – conjugated antibody and F-actin with TRITC-phalloidin (red). Representative images of *T. cruzi* and F-actin staining are shown **(F)** G strain and **(H)** Y strain. **(I,J,L)** FACS shows increased actin polymerization **(I)** associated with lower multiplication of the Y strain of *T. cruzi* **(J)** when rP21 treatment was performed. Representative images are shown **(L)**. Data are expressed as the mean ± standard deviation (SD) of experiments performed in triplicate. Significant differences were determined using Student's *t*-test (two-sided) test (a, c, e, g, i, j). Differences were considered significant when *p* < 0.05.

rP21 binds to CXCR4 receptor and promotes activation of PI3-kinase signaling pathway resulting in actin cytoskeleton remodeling (18). To determine the role of rP21 on actin cytoskeleton of host muscle cells, we added rP21 (rP21-treated) in the cell culture medium. Control cells did not receive rP21 treatment. At 96 h post-infection, we observed enhanced actin cytoskeleton polymerization in rP21-treated C2C12 cells as shown by the increase in the mean fluorescence intensity of TRITC-phalloidin-labeled actin depicted by microscopy (Figures 1A,B) and flow cytometry (Figures 1C,D). A similar effect was also observed by Teixeira et al. (20) in murine endothelial cell lines (T-end cells), suggesting that the role of rP21 on actin cytoskeleton polymerization applies to a variety of cell types.

Although the ability of rP21 to induce phagocytosis has been previously described (18), its role on parasite replication is still unknown. To investigate that, we infected C2C12 cells *in vitro* with the G or Y strains and treated them or not with rP21. As shown by fluorescence microscopy, rP21-treated C2C12 cells presented reduced number of parasites per cell (for both Y and G strains) and increased actin polymerization compared to control cells (Figures 1E–H). These results were confirmed by flow cytometry which showed a decreased expression of FITC-labeled *T. cruzi* and increased expression of TRITC-labeled F-actin in rP21-treated C2C12 cells (Figures 1I,J,L). It was seen by Teixeira et al. (23), that the treatment with rP21 of immortalized macrophages infected with strain Y, reduced the parasitic multiplication after 72 h. Here in our work, we seek to discover the role of the recombinant protein during the multiplication of *T. cruzi* in muscle cell line (C2C12 myoblasts), which resemble those naturally parasitized in the host organism. In addition, we seek to elucidate which mechanisms are involved in containing replication in these cells.

The importance of actin cytoskeleton during parasite replication has been previously demonstrated by other authors (24–26). AFAP-1L1 knockout cells, which showed lower actin polymerization, were found to have higher intracellular replication rate of strain *T. cruzi* (26). Previous studies have reported that a more rigid cytoskeleton retains the parasite within the host cell (24), while its disarrangement would be more conducive to the free intracellular replication of *T. cruzi* (25). These findings led us to propose that the enhanced actin polymerization induced by rP21 treatment forms a physical barrier that impairs the intracellular multiplication of *T. cruzi*.

**Figure 2:**
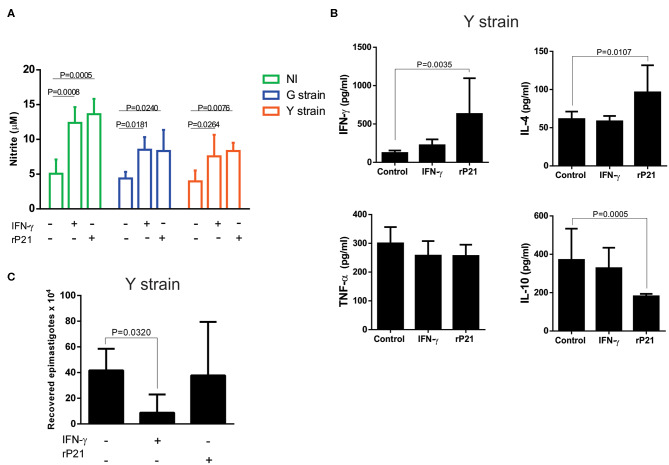
rP21 leads to increased production of NO, IFN-γ and IL-4 by myoblasts infected with the Y strain of *T. cruzi*, without causing the death of the parasite. **(A)** Infected or not (NI) myoblasts show a higher nitric oxide production when treated with rP21 or IFN-γ. **(B)** Supernatant of Y strain infected cells has higher levels of IFN-γ and IL-4 and lower levels of IL-10 when treated with rP21. **(C)** The ability of intracellular amastigotes recovered from rP21-treated myoblasts to differentiate into epimastigotes was not altered. Data are expressed as mean ± standard deviation of experiments performed in triplicate. Significant differences were determined using one-way ANOVA and Tukey's multiple comparisons test (a, b, c). Differences were considered significant when *p* < 0.05.

In addition to the physical barrier of F-actin, other mechanisms triggered by rP21 could also potentially affect the parasite multiplication. For example, rP21 may trigger cell activation and production of inflammatory molecules that lead to parasite death. To examine this hypothesis, we evaluated the levels of nitric oxide (NO) in the supernatant of uninfected (NI) and infected (G or Y strains) C2C12 cells, treated or not with rP21 or recombinant interferon-gamma (IFN-γ). Our results showed that the level of NO in the supernatant of both uninfected and infected cells treated with IFN-γ or rP21 is higher compared to the untreated cells (Figure 2A).

Furthermore, treatment of Y strain-infected C2C12 cells with rP21 increased the production of IFN-γ and IL-4 while it decreased the production of IL-10 compared to the non-treated (control) cells (Figure 2B). The ability of rP21 to induce IL-4 production has already been demonstrated in an *in vivo* model devoid of infection by Teixeira et al. (19). The duality in the profile of inflammatory molecules prevents us from classifying the rP21 induced response as predominantly Th1 or Th2 pattern of immune response. We observed that cells treated with rP21 presented higher NO and IFN-γ production. Cytokine (IFN-γ) is known to induce transcription of the gene encoding the enzyme nitric oxide synthase (iNOS), which increases the production of nitric oxide (27, 28). The administration of IFN-γ to mice in the acute phase of *T. cruzi* infection, results in decreased parasitemia and animal mortality (29), while treatment with nitric oxide inhibitors increases parasitemia and mortality rate of infected mice (30, 31). Cardiomyocytes produces several chemokines during *T. cruzi in vitro* infection as MCP-1 and MIP-1a, which plays important role in the uptake and killing of intracellular parasites by inducing NO synthase activation and increasing NO production (32–34).

The activation of inflammatory/anti-inflammatory molecules together with nitric oxide production could result in the death of the intracellular parasite. Therefore, we verified the viability of recovered intracellular amastigotes. Surprisingly, treatment with rP21 did not lead to decreased viability neither reduced the ability of the parasite to differentiate into epimastigotes, as shown in Figure 2C. Overall, these results indicate that despite the increased production of IFN-γ and nitric oxide by rP21-treated cells, amastigote did not suffer from its viability and ability to differentiate into other evolutionary forms. Despite the consensus on the protective effect of the IFN-γ cytokine during infection by *T. cruzi*, it has been reported that the parasite, once inside the cell, can antagonize the STAT1 transduction signal, selectively promoting the dephosphorylation of serine 727, decreasing its role in inhibiting intracellular replication and to promoting parasite death (35).

Epimastigotes incubation with rP21 protein had G1 phase prolongated while the synthesis phase S decreased (23). Furthermore, the role of rP21 in arresting the cell cycle is specific to *T. cruzi*, and no effect was observed on the species *L. (L) amazonensis*. When treating amastigotes with benznidazole, it appears that the largest proportion of the parasites are in the G1 phase, slowly returning to the normal replicative state after drug withdrawal. This plasticity of modulating the cell cycle in the face of stressful situations in the environment shows that *T. cruzi* has a sophisticated sense of response to external stimuli, being an important form of parasite persistence in mammalian host tissue (36).

In this context, we believe that the role of rP21 in decreasing intracellular parasite replication is due to a set of factors, in which the cytoplasmic physical barrier would act due to the more polymerized actin cytoskeleton, as well as the induction of immune molecules production by host cell. Both mechanisms would act concomitantly, controlling the replication and shifting the parasite cell cycle. However, the mechanisms that would explain such phenomena and the way they relate still need to be clarified.

### *In vivo* rP21 Treatment Reduces Parasite Load in Heart Tissue

Next, we evaluated the *in vivo* effects of rP21 protein in acute experimental infection with *T. cruzi*. BALB/c mice were subcutaneously (s.c) infected with two different doses, 10^6^ and 10^5^, of the Y strain trypomastigotes followed by treatment (s.c) with PBS or rP21 (100 μg/animal) on days 0, 5 and 15 post-infection.

**Figure 3:**
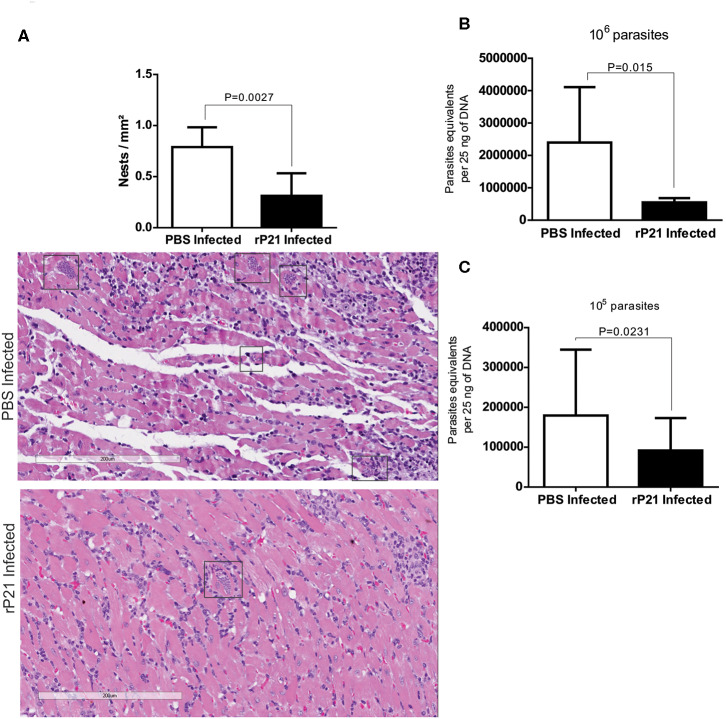
*In vivo* rP21 treatment reduces parasite load in heart tissue. **(A)** Treatment with recombinant protein decreases amastigote nests in cardiac tissue. Representatives images with amastigotes nests highlighted in rectangles. **(B,C)** qPCR quantification shows reduced parasite load in the hearts of 10^6^ and 10^5^ *T. cruzi*-infected mice that were treated with rP21. Data are expressed as mean ± standard deviation of experiments performed in triplicate. Significant differences were determined using Student's *t*-test (two-sided) (b) and Mann-Whitney test (c). Differences were considered significant when *p* < 0.05.

Histological heart samples from mice infected with 10^6^ parasites and treated with rP21 showed a significant reduction in the number of amastigote nests compared to samples from infected mice treated with PBS (Figure 3A). Tissues from mice treated with rP21 presented reduced parasite load independently of the inoculum dose (Figures 3B,C).

Taken together, these results corroborate with our *in vitro* multiplication assays and strongly suggest that P21 may be an important factor for intracellular confinement of *T. cruzi*, which contributes to its ability to establish a chronic infection. Decreased parasite burden in infected groups receiving rP21 treatment corroborate our *in vitro* findings reported here. A similar result was observed by Teixeira et al. (23), who evaluated the treatment with rP21 during chronic experimental infection with *T. cruzi*, observing a 50% reduction in the parasitic load (23). Such study used the paws as a route of infection and treatment, in addition to having carried out the treatment with rP21 every 72 h, for 6 weeks.

### Recombinant P21 Protein Reduces Cardiac Tissue Damage

During *T. cruzi* infection, trypomastigotes access the myocardium by invading the endothelium and vascular interstitial areas. The damage caused to the extracellular matrix by parasitic enzymes, contributes to the host's inflammatory response. Tissue damage and remodeling is characterized by structural changes associated with inflammation, necrosis, hypertrophy, ventricular dilation, and functional disorders, which are conditions seen in chagasic patients (37).

We achieve the qualitative analysis of cardiac tissue in different groups of mice. Table 1 shows the different variables analyzed according to intensity, where it was seen that the inflammatory infiltrate (pointed by black arrows) was intense (+ + +) in the group infected with 10^6^ and treated with PBS (Supplementary Figures 1C,E), moderate (++) in 10^6^ infected group treated with rP21 (Supplementary Figures 1D,F), moderate in 10^5^ infected group treated with PBS (++) (Supplementary Figure 1G) and mild (+) not infected with 10^5^ treated with rP21 (Supplementary Figure 1H). Thus, tissue destruction in infected with 10^6^ trypomastigotes groups was higher in PBS treatment compared to those which received rP21.

**Table 1:** Qualitative analyses of alterations in heart tissue.

**Groups**	**Inflammatory infiltrate**	**Tissue damage**	**Epicardium calcification**	**Nests**
PBS Control	–	–	–	–
rP21 Control	–	–	–	–
PBS Infected - 10^6^	+ + +	+ + +	++	+ + +
rP21 Infected - 10^6^	++	+	+	++
PBS Infected - 10^5^	++	+	–	+
rP21 Infected - 10^5^	+	+	–	–

*The rP21 treatment reduces cardiac inflammatory infiltrate, tissue damage, epicardium calcification and the number of amastigotes nests compared to infected control group*.

**Figure 4:**
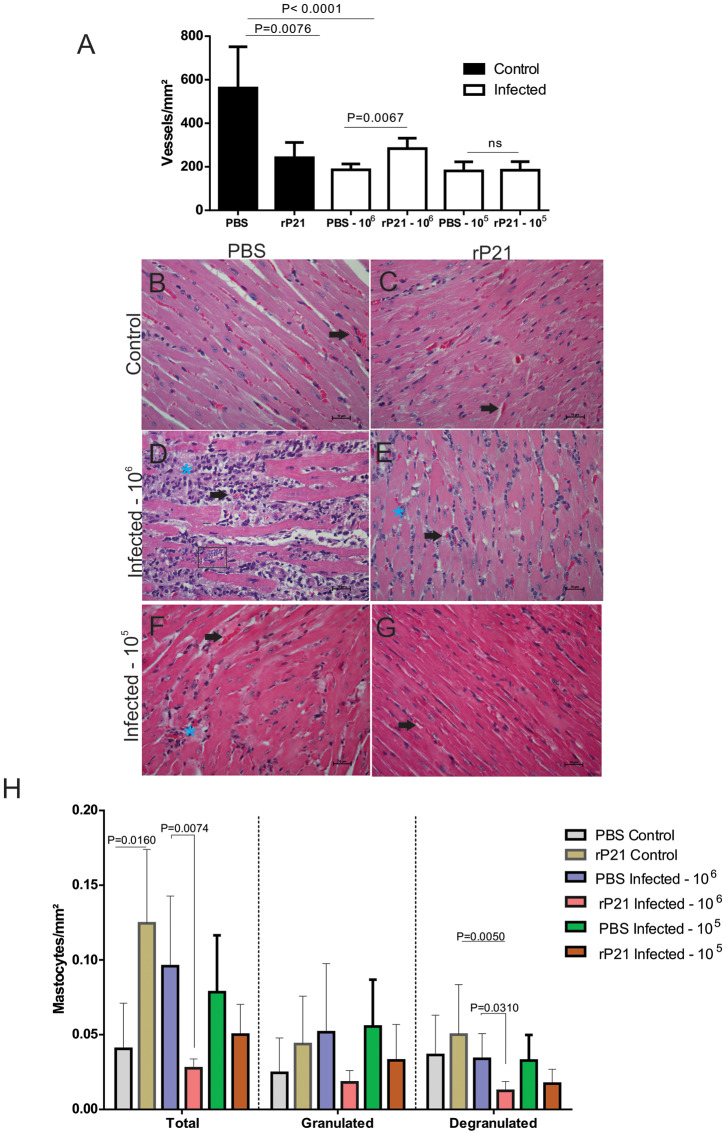
rP21 preserves angiogenesis of the heart and decreases the presence of mast cells in animals infected with *T. cruzi*. **(A)** Treatment of uninfected mice with rP21 reduces the amount of cardiac blood vessels. However, in mice infected with 10^6^ parasites and treated with rP21 (rP21-infected), the vascular architecture was preserved when compared to infected mice treated with PBS (PBS-infected). Representative images of H&E staining of heart tissues are shown with black points indicating blood vessels and blue asterisks inflammatory infiltrate areas (400 x magnification). **(B)** uninfected control and PBS treated. **(C)** uninfected animal treated with rP21. **(D)** infected with 10^6^ trypomastigotes and PBS treated. **(E)** infected with 10^6^ trypomastigotes and rP21treated. Infected animals with 10^5^ trypomastigotes and PBS **(F)** and rP21 **(G)** treated. **(H)** rP21 decreases the recruitment of mastocytes in cardiac tissue from mice infected with 10^6^ parasites. Data are expressed as mean ± standard deviation of experiments performed in triplicate. Significant differences were determined using Kruskal–Wallis and Dunn's multiple comparisons test (a, h). Differences were considered significant when *p* < 0.05.

Teixeira et al. (19) reported the anti-angiogenic role of rP21 in an *in vivo* sponge implant model. To further investigate the effect of rP21 treatment in cardiac tissue damage, we evaluated angiogenesis, the cell types constituting the inflammatory infiltrate and tissue fibrosis. Our findings that rP21 treatment in uninfected mice reduced the number of blood vessels in cardiac tissue confirmed Teixeira et al. results (Figure 4A). Although the number of vessels in mice infected with a high dose of parasite and treated with rP21 is higher than in those treated with PBS, they are not different from the uninfected mice treated with rP21 (Figures 4A,C,E). This suggests that the effect of rP21 treatment in the number of vessels was not affected by the high parasite load. In murine endothelial cells (EC), rP21 promotes the inhibition of EC proliferation, overexpression of the anti-angiogenic factor sFlt-1, and down-regulation of molecules with pro-angiogenic activity like Flt-1, ezrin, AFAP1, AFAP1L1, and moesin. These findings support the anti-angiogenic function of rP21 already reported in several studies (20).

Mastocytes have been implicated in cardiovascular dysfunctions, such as ischemic heart disease, experimental myocardial infarction, myocarditis, heart failure, transplant-related fibrosis, and hypertensive heart disease (19, 38–41). *In vitro* and *in vivo* studies have shown the presence of mastocytes associated with cardiac *T. cruzi* infection (42). Here, the mast cells count was significantly lower in the rP21-10^6^ infected mice (Figure 4H) compared to PBS-10^6^ infected mice, and it was associated with less inflammatory infiltrate (Table 1). These results support the modulatory role of rP21 during acute experimental infection with *T. cruzi*.

**Figure 5:**
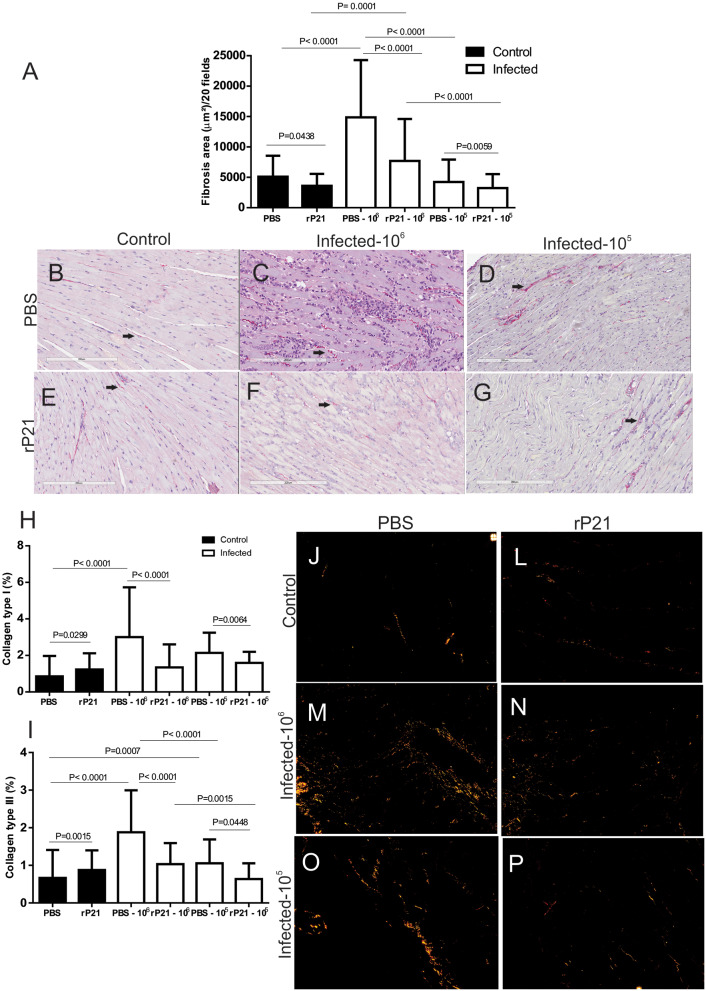
rP21 decreases fibrosis and levels of collagen type I and III in cardiac tissue. **(A)** Total heart collagen quantification shows that treatment with recombinant protein resulted in a decrease in total fibrosis area and in the percentage of type I **(H)** and type III **(I)** collagen in cardiac tissue. Representative images are shown as **(B,J)** uninfected control and PBS treated; **(E,L)** uninfected animal treated with rP21; **(C,M)** infected with 10^6^ trypomastigotes and PBS treated; **(F,N)** infected with 10^6^ trypomastigotes and rP21treated; **(D,O)** infected animals with 10^5^ trypomastigotes and PBS treated; **(G,P)** infected animals with 10^5^ trypomastigotes and rP21. Data are expressed as mean ± standard deviation of experiments performed in triplicate. Significant differences were determined Kruskal–Wallis and Dunn's multiple comparisons test (a, h, i). Differences were considered significant when *p* < 0.05.

Analysis of collagen fibers from cardiac tissue showed that treatment with rP21 decreased the fibrosis area, independent of the parasite dose used for infection (Figure 5A), with a significant reduction in type I and type III collagen (Figures 5H,I). The fibrosis area found in mice infected with 10^5^ parasites was smaller than in groups infected with 10^6^ parasites (Figure 5A). Such relationship was also observed in the measurement of type III collagen (Figure 5I). During chronic experimental infection with *T. cruzi*, treatment with rP21 for 42 days every 72 h (~14 times), resulted in a larger area of fibrosis associated with fewer vessels (23). Here, only three applications of rP21 were performed during the 21 days of infection, which leads us to conclude that the effect of rP21 *in vivo* during infection by *T. cruzi*, depends on the duration of the infection and the treatment regimen used.

Here we saw that the group of PBS-treated 10^6^-infected animals had higher levels of total and degranulated mastocytes, along with a larger area of fibrosis, and fewer blood vessels than rP21-infected and infected animals (Figures 4, 5). Such relationship between mastocytes, fibrosis, and destruction of vascular architecture has been previously seen in other studies. Chronic chagasic patients had a higher percentage of collagen correlated with high mastocytes density and reduced number of blood vessels in the heart (43, 44). This phenomenon is partially explained by the fact that mastocytes release chemical mediators, which stimulate the inflammatory process and collagen production (45, 46). The lower density of blood vessels in the group infected with 10^6^ and PBS treated, therefore, is possibly related to the fact that fibrosis predisposes to endothelial cell apoptosis, favoring regression of blood vessels (47).

Thus, the qualitative analysis of the cardiac tissue of the different groups shows that there is greater preservation of tissue architecture in animals infected and treated with rP21 protein, regardless of inoculum load. This tissue preservation appears to be related to the containment of parasitic replication, where *T. cruzi* confined in an intracellular environment would result in less stimulation of the immune response and, consequently, less damage to the host. By staying within the heart fibers, the parasite would be less prone to attack by the immune system, which would result in the perpetuation of infection.

### rP21 Stimulates the Simultaneous Production of IFN-γ and IL-10 *in vivo*

Current literature suggests that immune response developed by the infected mammalian host impacts disease progression and clinical manifestations (48). Resistance to *T. cruzi* infection has been associated with production of pro-inflammatory cytokines IL-12 and IFN-γ, and with local production of RANTES, MIP-1α, MIP-1β and MCP-1. These cytokines activate the production of nitric oxide by macrophages which is responsible for the elimination of the parasite (19, 49).

**Figure 6:**
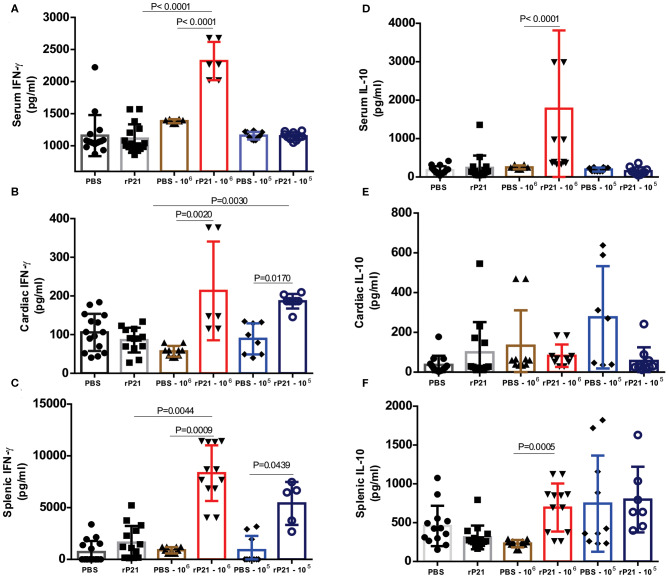
Mice treated with rP21 presented higher levels of IFN-γ and IL-10. **(A)** Infected mice treated with rP21 show higher levels of IFN-γ in serum, **(B)** heart and **(C)** spleen. **(D)** There are no significant differences in the levels of IL-10 in serum and **(E)** heart of infected mice treated with PBS or rP21, but the levels are up-regulated in **(F)** splenic tissue of rP21-treated mice. Data are expressed as mean ± standard deviation of experiments performed in triplicate. Significant differences were determined using Kruskal–Wallis and Dunn's multiple comparisons test. Differences were considered significant when *p* < 0.05.

To assess the immune profile triggered in mice infected or not with *T. cruzi* and treated with rP21 or PBS, we measured the levels of IFN-γ, IL-4, IL-10 and TNF-α cytokines in serum, heart and spleen. rP21 treatment led to a significant increase in the levels of IFN-γ in serum and splenic and cardiac tissues from mice infected with 10^6^ parasites (Figures 6A–C). Levels of IL-10 were increased in serum and spleen of mice infected with 10^6^ parasites, but not in their cardiac tissue (Figures 6D–F). There was no significant difference in the levels of IL-4 and TNF-α among the analyzed groups (Supplementary Figure 2). The exception was the IL-4 levels in the spleen which were lower in the groups infected with 10^6^ parasites vs. those infected with 10^5^ parasites (Supplementary Figure 2), regardless of the treatment performed.

Teixeira et al. (19) observed an up-regulation of IL-4 induced by rP21 in sponge implants. Here, we confirmed the increase in IL-4 production only with *in vitro* experiments (Figure 2B), but no significant differences were observed *in vivo*. This inconsistency may be due the experimental model or to differences in the time of infection evaluated *in vitro* and *in vivo* (96 h vs. 21 days). Therefore, it is possible that an up-regulation of IL-4 may be observed *in vivo* if measurements are performed at earlier time points. In addition, *in vivo* infection is more complex than *in vitro* infection, involving other components that act together to establish an immune response.

Interferon-gamma (IFN-γ) was up-regulated in both *in vitro* and *in vivo* studies. It has been *in vivo* demonstrated that cardiomyocytes are a potential source of cytokines, chemokines, and NO (33). TNF-α and IFN-γ cytokines activates NO production through iNOS, which acts by modulating the production of chemokines in an autocrine and / or paracrine manner and amplify the inflammatory response. NO production in the mice *T. cruzi* – infected myocardium, controls parasite growth and modulates the synthesis of several chemokines, such as CCL2, CCL3, CCL4, CCL5, and CXCL2 (37). Lykens et al. demonstrated *in vivo* that the action of the IFN-γ cytokine in controlling infections by various parasitic protozoa, including *T. cruzi*, is dependent on direct interaction with macrophages. Using macrophages derived from transgenic mice that had IFN-γ insensitive macrophages (MIIG), it was observed the unable of this cell in produce NO and kill *T. cruzi* and *Leishmania major*. Infected transgenic animals had uncontrolled parasitemia and a high mortality rate, despite having appropriate IFN-γ production. Meanwhile, MIIG mice were able to control infection with the lymphocytic choriomeningitis virus. Thus, in order to control infection by *T. cruzi*, there must be a direct interaction of the IFN-γ cytokine with macrophages (50).

*In vivo*, we found up-regulation of both IFN-γ and IL-10, probably due to the regulatory balance of inflammation. It is postulated that IL-10 signaling does not compromise the cardiac tissue of patients and leads to the indeterminate form of the disease, while the cardiac form is induced and maintained by an inflammatory cytokine profile with IFN-γ and TNF-α playing a pivotal role (51, 52). Thus, treatment with rP21 led to a balanced level of inflammation, which is probably related to the decrease in tissue damage found in mice treated with this protein (Table 1).

As previously reported, groups of infected animals receiving rP21 treatment had a smaller area of fibrosis compared to uninfected controls. Such a phenomenon can be correlated with the higher presence of IFN-γ in the cardiac tissue of these animals. Studies show that the presence of interferon-gamma is related to the inhibition of collagen synthesis in mesenchymal cells (53–55). This response involves the transcriptional repression of collagen genes as COL1A1 and COL1A2, in addition to using ERK1/2 kinase and C/EBPβ signaling pathway to down-regulate collagen expression (56, 57). Thus, in the PBS infected−10^6^ group, the greater presence of mastocytes (Figure 4H) and other cells in the inflammatory infiltrate would generate tissue damage in the heart (Supplementary Figures 1C,E). Meanwhile, those receiving rP21 as treatment had a higher IFN-γ stimulus that inhibits collagen synthesis, and consequently fibrosis, which combined with the simultaneous presence of IL-10 decreases the heart damage caused by the exacerbated inflammation.

### P21 Is Most Expressed in Different Stress Conditions

**Figure 7:**
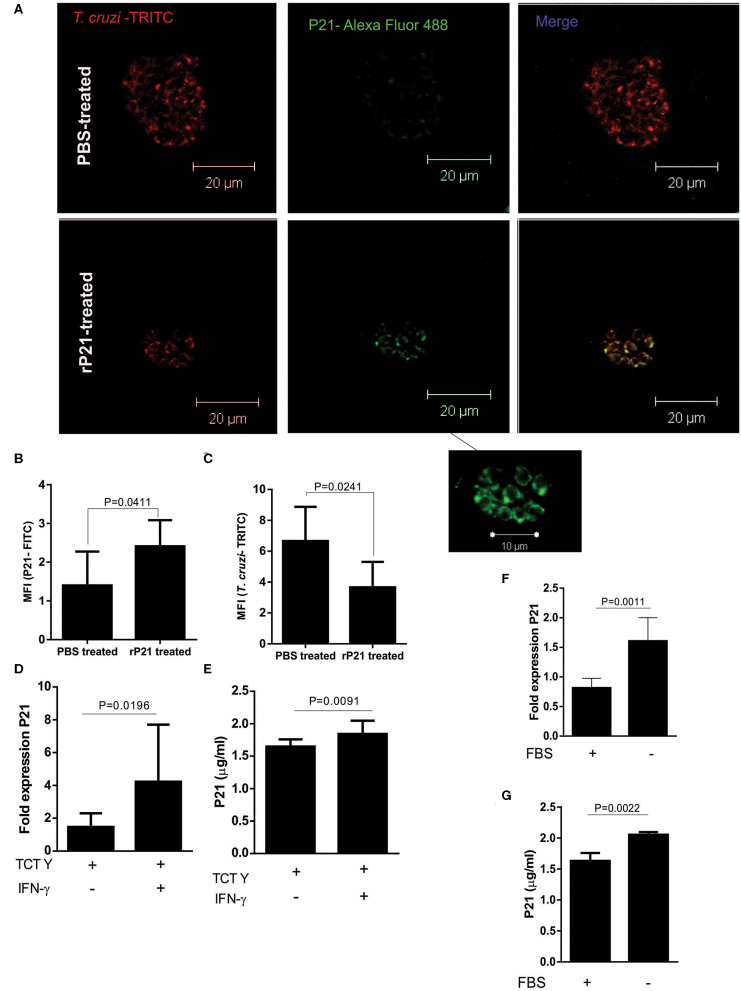
rP21 as a soluble factor acts as an inducer of *T. cruzi* dormancy. **(A)** Amastigotes from cardiac nests qualitatively produced more native P21 when mice received treatment with the recombinant protein. **(B)** P21 quantification shows a higher presence of this protein in amastigote nests of mice treated with the recombinant protein in contrast to the lower amount of **(C)** *T. cruzi* found in these nests. **(D)** P21 was highly expressed and produced **(E)** when infected cells were exposed to IFN -γ induced stress. Epimastigotes subjected to nutritional starvation **(F)** expressed and **(G)** produced more native P21. Data are expressed as mean ± standard deviation of experiments performed in triplicate. Significant differences were determined using unpaired *t*-test and Student's *t*-test (two-sided) (c, e, f) and Mann-Whitney test (b, d, g). Differences were considered significant when *p* < 0.05.

To analyze whether cardiac nests of amastigotes produce native P21, we performed P21 labeling on the cardiac tissue of mice infected with *T. cruzi*. We observed a higher expression of native P21 in cardiac nests from mice that received recombinant protein as treatment (Figures 7A,B). We also quantified TRITC-labeled *T. cruzi* in the nests and found a higher fluorescence intensity in PBS-treated than in rP21-treated mice (Figure 7C), corroborating with the higher parasite load described earlier (Figures 7D,E). To determine if recombinant protein accumulated in cardiac tissue and could interfere with our analyzes, we performed P21 labeling on the heart of rP21-treated uninfected animals (data not shown), which showed no presence of this molecule in the samples analyzed.

These findings led us to investigate whether higher amounts of P21 could be related to tissue stress induction. As previously mentioned, tissues from mice treated with the recombinant protein presented high levels of IFN-γ (Figures 6A–C). IFN-γ may induce a stressful environment for parasite survival, which could lead to a higher release of P21 by intracellular amastigotes.

To evaluate whether the expression and secretion of P21 increases under unfavorable conditions for the parasite, we infected C2C12 cells with the Y strain and treated with IFN-γ. Both ELISA performed with cell extract and RT-PCR showed that the presence of IFN-γ increased the expression of P21 by the intracellular parasites (Figures 7D,E). These results suggest a positive regulation of P21 in the cardiac tissue of mice presenting higher amounts of IFN-γ. We also tested if P21 could be upregulated when epimastigotes are submitted to nutritional stress, mimicking the phenomenon observed in the digestive tract of the triatomine. For this purpose, we cultured epimastigotes in FBS-free medium for up to 48 h. Our results showed a higher expression of P21 by parasites incubated in the absence of FBS (Figures 7F,G).

Recently, a new mechanism of *T. cruzi* survival has been described. Authors proposed the ability of the parasite to remain in a dormancy state when facing a stressful environment (58). This mechanism is well-studied in bacteria and plays a critical role in their adaptation to changes in the environment.

*T. cruzi* dormancy seems most likely associated with evasion of host immune responses, the major stressor of amastigotes in vertebrate hosts. Since immune response may lead to complete resolution of infection (58, 59), the dormancy could facilitate parasite persistence in the host. Once the parasite invades a cell and decreases or interrupts its replication, it is not detected by the immune system. One possibility is that the parasite returns to its normal replicative rate after a certain period to allow the continuity of its life cycle. Alternatively, dormancy might be a consequence of *T. cruzi* biology rather than an evolutionarily selected process for low-level persistence. In fact, the mechanisms for entering and exiting the dormant state by *T. cruzi* is completely unknown (58).

Dumoulin and Burleigh (36) reported the plasticity of intracellular *T. cruzi* amastigotes to dynamically adjust their proliferation rates in response to different stressors (36). On their analysis, after transient nutrient withdrawal, chemical inhibition of glycolysis or respiration, there was reversible slowing of intracellular amastigote proliferation with increased proportion of parasites in the G1 phase of cell cycle. Parasites resumes growth when stressful conditions have been removed. This study related the plasticity of *T. cruzi* with the ability to hide in the resident tissue when exposed to the drug (benznidazole). Plasticity is a distinct mechanism from spontaneous dormancy (58) and indicates a sophisticated pathway of detection and response coupled with cell cycle regulation as an important pathway for long-term persistence of *T. cruzi* in mammalian hosts (36).

Our results showed that rP21 decreased *T. cruzi* replication, without causing parasite death. P21 uses several mechanisms of the cellular and immunological host's machinery in order to confine this protozoan in the intracellular environment, leading the parasite to a latent state, which may indicate an evolutionary mechanism of survival developed by the parasite. Thus, when subjected to different stress conditions, the protozoan produces more P21, which induces *T. cruzi* latency in the host organism, enabling the protozoan to evade the host immune system The highest production of P21 by *T. cruzi* under stressful environment may be related to the newly little characterized mechanisms of plasticity (36) or dormancy (58) of *T. cruzi*. Moreover, related to the vascular changes, we demonstrated in the present work that P21 was able to modulate the production of cytokines and the inflammatory process. Taken together, we believe that *T. cruzi* P21 has relevance on the onset and progression of CCC and may be a potential target for the development of P21 antagonist compounds to treat chagasic cardiomyopathy. In this sense, we are currently addressing synthetic peptides based on sequences expressed by bacteriophages (phage display), that have binding affinity for P21 and potential inhibition of its function.

## Data Availability Statement

Data supporting this study can be found in the Supplementary Material. A complete description of the methods and the datasets generated for this study are available upon request to the corresponding author.

## Ethics Statement

Maintenance and care of animals complied with the guidelines of the Ethics Committee for the Use of Animals (CEUA) from the Universidade Federal de Uberlândia. This study was approved by CEUA-UFU, with protocol number 087/15.

## Author Contributions

FM, MSa, and CS: conceptualization. FM, MSa, and CS: methodology. FM, MSa, and CS: validation. FM and MSa: formal analysis. FM, MSa, JS, AS, BB, MC, PT, ST, RB, TTe, CR, NS, RO, LF, ML, JM, and MSi: investigation. RZ, TTo, MSi, CO, and CS: resources. FM, MSa, CO, and CS: writing—original draft preparation. FM, MSa, CO, and CS: writing—review and editing. FM, MSa, CO, and CS: visualization. CS: supervision, project administration, and funding acquisition.

## Conflict of Interest

The authors declare that the research was conducted in the absence of any commercial or financial relationships that could be construed as a potential conflict of interest.

## Supplementary Material

Supplementary Figure 1 Representative images of cardiac histological changes caused by infection by 10^6^ or 10^5^ trypomastigotes of *T. cruzi* and the effect of treatment or not with rP21. Mice were infected with 10^6^ or 10^5^ trypomastigotes of *T. cruzi* and treated or not with rP21. After 21 days, animals were euthanized, and heart fragments analyzed. **(A)** uninfected control. **(B)** uninfected animal treated with rP21. Black arrows point to inflammatory infiltrate. **(C)** infected with 10^6^ trypomastigotes and PBS treated. The white arrow point calcification area (100 x magnification). **(D)** infected with 10^6^ trypomastigotes and rP21treated (100 x magnification). **(E)** PBS-10^6^ group with nest highlighted in rectangle (400 x magnification). **(F)** rP21-10^6^ group (400 x magnification). Infected animals with 10^5^ trypomastigotes and **(G)** PBS and **(H)** rP21 treated (400 x magnification).

Supplementary Figure 2 IL-4 and TNF-α levels are not altered with rP21 treatment. **(A)** IL-4 levels in serum and **(B)** heart are not altered between groups analyzed. **(C)** The splenic levels of IL-4 are higher in mice infected with 10^5^ than with 10^6^ parasites. There was no difference in the levels of TNF-α in **(D)** serum, **(E)** heart, and **(F)** spleen from the different groups. Data are expressed as the mean ± standard deviation of experiments performed in triplicate. Significant differences were determined using Kruskal-Wallis and Dunn's multiple comparisons test. Differences were considered significant when *p* < 0.05.
