# Supplementary figures and images for: The Recombinant Form of Trypanosoma cruzi P21 Controls Infection by Modulating Host Immune Response
Source: Front Immunol. 2020 Jun 5;11:1010. doi: 10.3389/fimmu.2020.01010 (PMC7325895; doi:10.3389/fimmu.2020.01010)

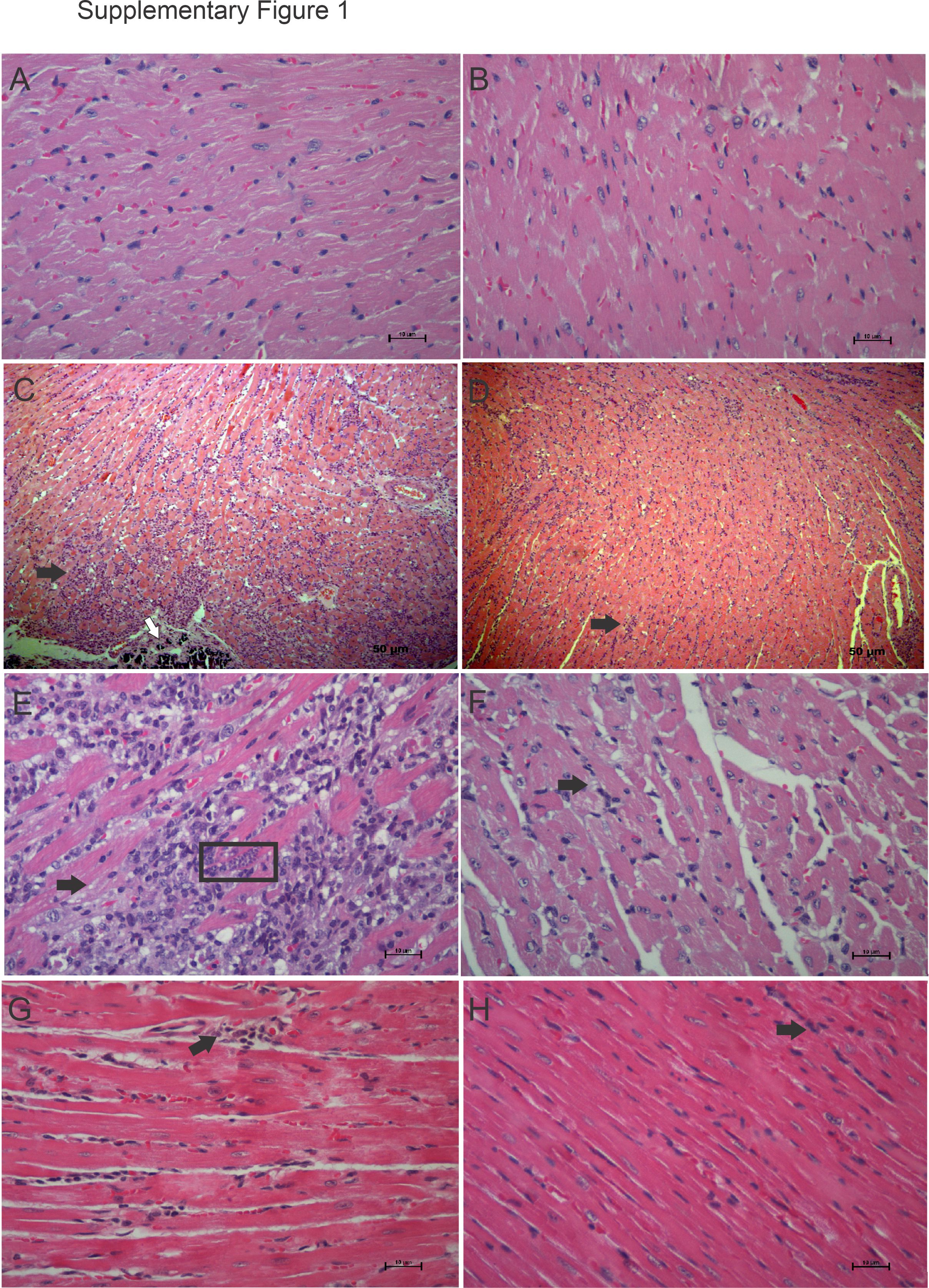

Supplement: Supplementary Figure 1 — Representative images of cardiac histological changes caused by infection by 106 or 105 trypomastigotes of T. cruzi and the effect of treatment or not with rP21. Mice were infected with 106 or 105 trypomastigotes of T. cruzi and treated or not with rP21. After 21 days, animals were euthanized, and heart fragments analyzed. (A) uninfected control. (B) uninfected animal treated with rP21. Black arrows point to inflammatory infiltrate. (C) infected with 106 trypomastigotes and PBS treated. The white arrow point calcification area (100 x magnification). (D) infected with 106 trypomastigotes and rP21treated (100 x magnification). (E) PBS-106 group with nest highlighted in rectangle (400 x magnification). (F) rP21-106 group (400 x magnification). Infected animals with 105 trypomastigotes and (G) PBS and (H) rP21 treated (400 x magnification). [file Image_1.jpg]

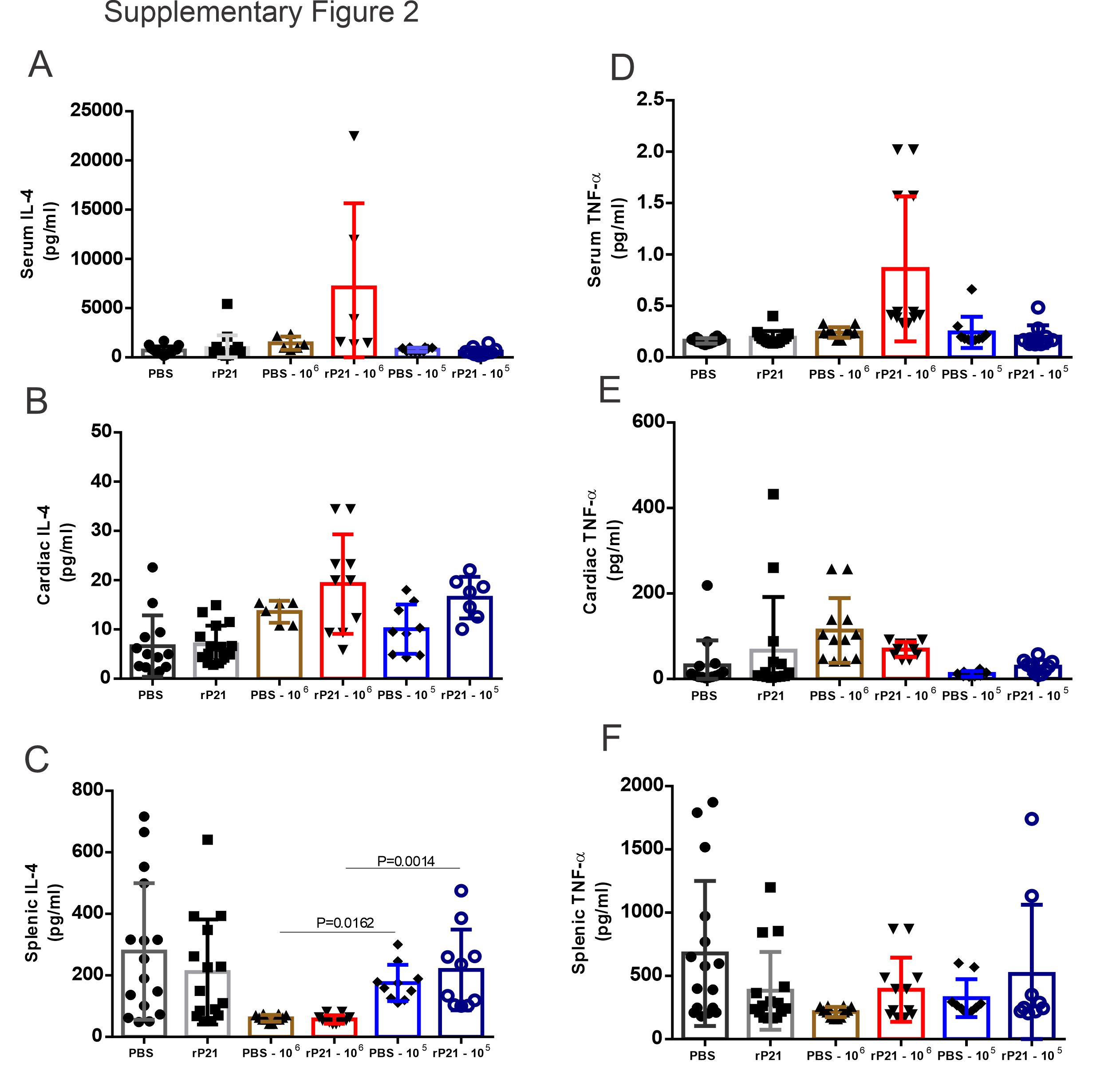

Supplement: Supplementary Figure 2 — IL-4 and TNF-α levels are not altered with rP21 treatment. (A) IL-4 levels in serum and (B) heart are not altered between groups analyzed. (C) The splenic levels of IL-4 are higher in mice infected with 105 than with 106 parasites. There was no difference in the levels of TNF-α in (D) serum, (E) heart, and (F) spleen from the different groups. Data are expressed as the mean ± standard deviation of experiments performed in triplicate. Significant differences were determined using Kruskal-Wallis and Dunn's multiple comparisons test. Differences were considered significant when p < 0.05. [file Image_2.jpg]
